# Gradient NMR Method for Studies of Water Translational Diffusion in Plants

**DOI:** 10.3390/membranes11070487

**Published:** 2021-06-29

**Authors:** Alexander Anisimov

**Affiliations:** Kazan Institute of Biochemistry and Biophysics, Russian Academy of Sciences, 420111 Kazan, Russia; anisimov@kibb.knc.ru

**Keywords:** gradient spin-echo NMR method, water diffusion, plant roots

## Abstract

The review of a retrospective nature shows the stages of development of the spin-echo NMR method with constant and pulsed gradient of the magnetic field (gradient NMR) for the study of water diffusion in plant roots. The history of the initial use of gradient NMR for plants, in which it was not possible to experimentally confirm the bound state of water in cells, is described. The work presents the main ideas on which the technology of measuring diffusion by the spin-echo NMR method is built. Special attention is paid to the manifestations and record of the restricted diffusion phenomenon, permeability of membranes, along with the finite formulae used in real experiments. As examples, it gives the non-trivial results of studies of water transfer in roots through the symplastic system, from cell to cell through intercellular contacts with plasmodesmata, through aquaporins, transfer under the influence of changes in external pressure, and the composition of the gas atmosphere.

## 1. Introduction

Despite the considerable amount of information on the condition and transport of water in plants, overall information on certain sections and even key issues remains limited. One reason lies in the limited number of methods adequate for the heterogeneous dynamic structure of living systems. In this regard, the spin-echo NMR method brings a fresh stream into the informative flow on the problem of water exchange, being largely adequate both for the problem and the object of research. Its adequacy is ensured through sensitivity to thin atomic molecular movements with the ability to work with cells, segments, and intact objects without violating their structure, and the ability to measure direct characteristics of transfer, such as the coefficients of diffusion, permeability, water flow rate, etc.

## 2. Prerequisites for Applying Spin-Echo NMR Method to Studies of Water Condition and Transport in Plants

### 2.1. The Prehistory of the Problem of the Condition of Water in the Cells

At one time, the uniqueness of the properties of water did not fail to attract the attention of physiologists in explaining the peculiarities of manifestations of cellular metabolism. In the 1960s, plant physiologists had to choose between two hypotheses about the condition of water in cells. According to the first hypothesis, the structure of water in cells differs from bulk water in the direction of greater orderliness, where it is bound, structured water. According to the second hypothesis, the structure of water in the cell for the most part is similar to such bulk water and only a small part, which has contact with non-aqueous components of the cell, hydrate water, is bound. Both concepts had their adherents, but the most vivid, reasoned, revolutionary concept of bound water was actively developed and promoted by the famous American scientist Gilbert Ling, who presented it as an alternative to the prevailing views. He held the opinion that intracellular water is ordered in the form of a multilayer structure of polarized water [1]. One strong argument for the concept of bound water could be the data on the translational mobility of water in cells. It is logical for bound water to expect a reduced value of the coefficient of translational self-diffusion. In the late 1960s, a promising non-invasive method for measuring water self-diffusion emerged—an NMR spin echo method with a constant time, followed by a pulsed gradient of magnetic field (hereinafter gradient NMR).

Already, the first experiments showed that the self-diffusion coefficient of water in plant tissues is noticeably less than that of ordinary, bulk water [2,3]. It seemed that the direct experimental data for the hypothesis about the special structured state of water in cells were obtained. However, it was soon discovered that the reduction of the measured self-diffusion coefficient of water in cells is owed to the effect of restricted diffusion: Diffuse water molecules collide with membranes and other barrier structures of cells and run a smaller diffusion path than they could run without barriers. As a result, the authors [2,3] diametrically changed their point of view on the structure of water: The bulk of the water in the cells does not differ in structure from free, bulk water, except for a small part of it, which is bound by non-aqueous components of cells [4].

Although the gradient NMR method holds a special place among other methods, due to its non-invasiveness and sensitivity to thin molecular movements, in general, the NMR method failed to solve the problem of the structural state of water in cells. As a result, the focus of research shifted from the structural condition of water to a clearly more welcome physiological problem—water transport and its main components: Transport routes, driving forces, barrier and regulatory functions of non-aqueous structures, and signal transfer systems. [5]

The purpose of this work is to give an idea of the possibilities of using gradient NMR and examples of using it in water transport studies in plant objects.

### 2.2. The Main Structures That Determine the Transport of Water in Plant Tissues

**Figure 1:**
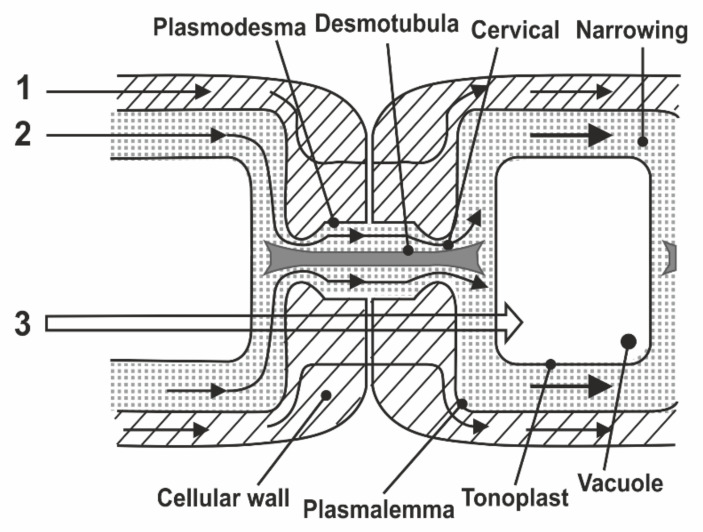
Scheme of water movement between root cells in the radial direction: (1) Apoplasmic way through the cell walls; (2) symplasmic way on plasmodesmata, cytoplasm; (3) cell-to-cell way through tonoplast, cytoplasm, plasmalemma, cell walls.

The global problem of water transport by signs of systemic differentiation of processes consists of three components: (1) Intracellular water metabolism, which includes the diffusion of intracellular water and the rotational movement of protoplasm; (2) near transport–transfer between adjacent cells and groups of cells; and (3) long-distance transport associated with mass flow of water between the organs of the plant. The most important classical example and the subject of study of intercellular transport is radial transport of water in roots. From the anatomical structure of the root, it follows that the intercellular transport of water can be carried out in three ways [6]: Through apoplast, which includes intercellular intervals, channels between microfibrils of cellulose of cell walls, and xylem vessels; through symplast, which includes internal contents of all cells united by intercellular channels with plasmodesmata into a single system; and through the transcellular–(transmembrane) pathway from cell to cell, with the intersection of the vacuole and with the egress of water through the plasmalemma into the intercellular space. The literature also uses the term cell-to-cell (from cell to cell), which refers to the combined transcellular + symplastic pathways, due to the experimental difficulties of their separate study [7], as shown in Figure 1.

## 3. The Development of Gradient NMR from the Constant Gradient Method to Pulse Method

As is known, when using the spin echo nuclear magnetic resonance (NMR) method, the study sample is placed in a magnetic field with a tension of B_0_, and emerging macroscopic magnetization M (0) deviates from the equilibrium position by the radio- frequency (RF) pulse of the resonant frequency—ω_o_, (ω_o_ = γ B_0_), where γ is gyromagnetic ratio for resonating nuclei [8]. To compensate for the static component of the distribution of Larmor frequencies due to the regular magnetic field gradient, which is a prerequisite for observing the spin echo signal, a sequence of two RF pulses—90° and 180° separated by an interval is used τ [8]. When the τ interval is varied, the amplitude of echo—M (2τ) decreases with time constant T_2_, due to the processes of spin–spin relaxation:

(1) $$M\;\left(2\tau \right)=\;M\;\left(0\right)\;\exp\;\left(─2\tau /{Т}_{2}\right)$$

In many spin-echo experiments, the time of spin–spin relaxation, T_2_, is one of the main required values.

Details of the calculation of the evolution of magnetization–echo signals depending on the parameters of the pulse sequence and special features of the studied samples are widely reported in the literature [9,10,11,12].

The first experiments that started the NMR spin echo method showed the need for reporting the phenomenon of diffusion of spins containing molecules [8]. The point is that the diffusion of molecules in the magnetic field gradient leads to a change in the frequency of the Larmor precession of nuclear spins. Changes in frequency lead to irreversible dephasing of the spin packet and, as a result, to the decrease in the amplitude of the echo signal.

The emphasis on this phenomenon in works [13,14,15,16] led to the announcement of a new method of studying translational displacements of spin-containing molecules—a gradient NMR.

There are two varieties of the gradient NMR: With the superposition of a constant and a pulse gradient of the magnetic field.

### 3.1. Constant Gradient Method

In the spin-echo NMR method with a constant gradient, a linear, time-constant gradient of the magnetic field with a value of $g_{0}$ is additionally superimposed on the standard laboratory magnetic field, which, in fact, acts as a spatial marker of the position of molecules. Quantitative estimation of the self-diffusion of molecules by the gradient NMR is based on recording the loss of phase coherence of spins due to their translational displacements in the magnetic field gradient.

The processes occurring with macroscopic magnetization under the action of a magnetic field gradient are described in detail in a series of original articles and reviews [13,14,15,16]. To obtain an explicit functional dependence of the echo amplitude on the parameters of the pulse sequence and the magnitude of the gradient, as a rule, either a solution of a system of equations for diffusion of the nuclear Bloch–Torrey magnetization [13] or a Douglas–McCall phase accumulation algorithm [14] is used.

In a Gaussian distribution of translational displacements of molecules, the echo amplitude M$\;\left(2\tau ,g_{0}\right)\;$for homogeneous liquid follows the expression [13,14]:

(2) $$M\;\left(2\tau ,g_{0}\right)=M\;\left(2\tau ,0\right)\exp\;\left(─\left(2/3\right)\gamma^{2}g_{0}^{2}\tau^{3}D\right)$$

where D is the self-diffusion coefficient, (2 τ− time of observation of diffusing molecule-diffusion time, which is limited to the magnitude T_2_).

The values M(2τ,0) characterize the change in echo amplitude due to spin–spin relaxation processes.

Primary information about the value D is usually obtained at a fixed diffusion time value, 2τ, from the dependence of the relative amplitude of the echo, factor R = M($\tau ,g_{0}$)/M(τ, 0), from the magnetic field gradient value$,\;g_{0}$ [13,14]:

(3) $$R=\exp-\frac{2}{3}\gamma^{2}g_{0}^{2}\tau^{3}\;D$$

The constant gradient method has been successfully applied for the study of the translational mobility of liquids with sufficiently high values of the self-diffusion coefficient [16,17,18,19].

However, with many advantages, the constant gradient method has drawbacks in applications to objects with low values of the self-diffusion coefficient, much less with short magnetic relaxation times. This is due to the limitations of 2 τ magnitude by the τ time of spin–spin relaxation and the limitation regarding the magnitude of the constant gradient of the magnetic field by the value exceeding which causes heterogeneous widening of the NMR absorption line. As a result, a part of the sample volume goes beyond the magnetic resonance frequency band. Nevertheless, there are examples of applying the constant gradient method through the use of the scattering field of a powerful magnet with the achievement of the gradient value up to 100 T/m and above, but the consequence of this gradient value is a significant reduction in the size of the sample [20].

### 3.2. Pulse Gradient Method

The principal possibility of using a significantly large-value gradient was realized in 1965 by Stejskal and Tanner [21], in the variant of a pulsed application of the magnetic field gradient—the so-called NMR spin-echo pulse gradient method.

**Figure 2:**
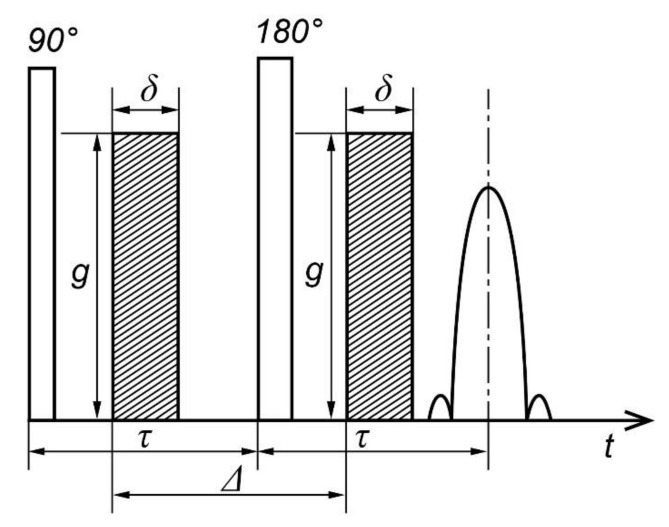
Two-pulse Hahn method with a pulsed magnetic field gradient (PGSE—pulse gradient spin-echo).

In the variant of the pulsed gradient NMR in the classical Hahn Method, for the pulses of the magnetic field gradient, the duration, δ, and the amplitude, g, are applied for a short time: The first one in the interval between 90° and 180° RF pulses, the second one between 180° and echo signal. Δ is the interval between them—the time of diffusion, as shown in Figure 2.

The first gradient pulse (GP) specifies the phase of the precession of spins according to their position at this point in time, and the second one serves as determining the phase shift over the diffusion time between GP.

Since RF and gradient pulses do not intersect in time, the amplitude of the latter is not limited from above and can reach significant values of about 200 T/m. As a result, the range of measured diffusion coefficients expands up to 10^−15^ m^2^/sec and it becomes possible to reduce the diffusion observation time, Δ, to a value of about a millisecond, which is limited by the minimal T_2_ magnitude and magnet system recovery time after the gradient pulse. In addition, in the pulse gradient method, the motion of spins within the interval Δ affects the echo amplitude with equal effectiveness, while in the constant gradient spin displacement method, 180° RF pulses are more effective [21]. The latter makes it difficult to analyze data in cases of dependence of the diffusion bias on the diffusion time.

**Figure 3:**
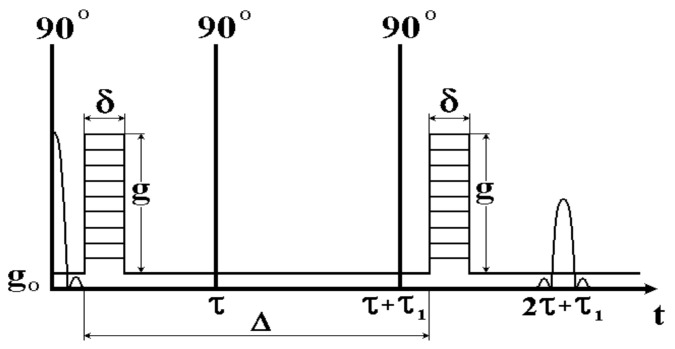
Sequence of stimulated echo with pulsed magnetic field gradient (PGSTE).

In order to extend the range of variation of diffusion time for samples where the spin-lattice (longitudinal) relaxation time, T_1_, is much greater than the spin–spin (transverse) relaxation time, T_2,_ which is often performed for biological objects, Tanner [22] realized the need to include gradient pulses in the three-pulse RF sequence of the stimulated echo, as shown in Figure 3. When the *τ*_1_ interval changes, the amplitude of the stimulated echo decays with the spin-lattice relaxation time Т_1._ For water of many biological objects, Т_1_ is much longer than the spin–spin relaxation time Т_2,_ and thus a greater range of variation of diffusion time, Δ, is achieved, while the value ∆ is limited from above by the value T_1._

The diffusion time in PGSTE, as in the previous PGSE case, is determined by the distance between the gradient pulses and varies by a change in *τ*_1_.

With the use of the Douglas–McCall phase accumulation algorithm [14] for the Gaussian distribution of translational displacements of molecules, Stejskal and Tanner obtained the expression of dependence of the relative echo amplitude M (Δ,g)/M (Δ, 0), factor R, from the parameters of the gradient pulses and the value of the self-diffusion coefficient-D, [21]:

(4) $$R=\exp-\gamma^{2}\delta^{2}g^{2}(\Delta -\frac{1}{3}\delta )D$$

The parameters of the gradient pulses enter (4) linearly in the form of factors and, therefore, if diffusion is not restricted, the value of *D* does not depend on what varies in the experiment: Amplitude, duration, or the interval between gradient pulses.

A significant range of gradient variation determines the successful application of the method to the study of diffusion in multiphase systems [23,24]. With no exchange, the R factor is presented by the sum of exponential functions:

(5) $$R=\overset{N}{\underset{k=1}{\sum }}p_{k}\exp\left(-\gamma^{2}\delta^{2}g^{2}D_{k}\;\Delta \right)$$

where $p_{k}$ are shares and $D_{k}$ are coefficients of diffusion of every component of the multiphase system.

With a quick exchange:

(6) $$R=exp-\left[\gamma^{2}\delta^{2}g^{2}\left(\overset{N}{\underset{k=1}{\sum }}p_{k}D_{k}\right)\Delta \right]\;$$

If the system is characterized by the distribution of diffusion coefficients, *P* (D), then:

$$R\left(g\right)=\int_{0}^{\infty }P\left(D\right)exp-\gamma^{2}\delta^{2}g^{2}D\left(\Delta -\delta /3\right)dD,$$

(7) $${\left.\frac{\partial \left[\ln R\left(g\right)\right]}{\partial \left[-\gamma^{2}\delta^{2}g^{2}\left(\Delta -\delta /3\right)\right]}\right|}_{g\to 0}=\overset{\infty }{\underset{0}{\int }}P\left(D\right)DdD=\bar{D}$$

where $\bar{D}$ is the average diffusion coefficient determined by the initial slope of diffusion attenuation.

For a relatively complex case of two-phase systems with interphase exchange, where molecules can be in two positions with an average lifetime of molecules in phases $\tau_{1},\tau_{2}$, with shares Р_1_ and Р_2_ and of coefficients of diffusion, $D_{1},D_{2}$, Karger [24] gives an expression for the R factor in the form of:

(8) $$R\left(\delta g,\Delta \right)=P_{1}^{\prime }exp\left[-{\left(\gamma \delta g\right)}^{2}\Delta D_{1}^{\prime }\right]+P_{2}^{\prime }exp\left[-{\left(\gamma \delta g\right)}^{2}D_{2}^{\prime }\Delta \right]$$

where D1,2′=12D1+D2+1/γδg2·1τ1+1τ2∓D2−D1+1γδg21τ2−1τ12+1γδg4τ1·τ2

$$P_{2}^{\prime }=\frac{1}{D_{2}^{\prime }-D_{1}^{\prime }}\left\{P_{1}D_{1}+P_{2}D_{2}-D_{1}^{\prime }\right\},\;P_{1}^{\prime }=1-P_{2}^{\prime }$$

where $P_{1}^{\prime }$,$\;P_{2}^{\prime },\;D_{1,2}^{\prime }$ are apparent experimentally measured values.

Without exchange:

(9) $$R\left(\delta g,\Delta \right)=P_{1}exp[-{\left(\gamma \delta g\right)}^{2}\Delta \;D_{1}]+P_{2}exp[-{\left(\gamma \delta g\right)}^{2}\Delta \;D_{2}]$$

For the diffusion of particles in narrow long pores whose diameter only slightly exceeds the diameter of diffusing particles, Levitt [25] obtained an expression for the probability of one-dimensional displacement of particles. In [26], the Levitt ratio is used to obtain the ratio for the *R* factor:

(10) Rδ,Δ,D0=exp−γ2δ2g2D0Δπρ212 

where $D_{0}$ is the diffusion coefficient for the case where there is only one molecule in the pore and ρ is the density of particles in the pore.

In a random pore orientation in space, averaging over the hemisphere gives the ratio for R [26]:

(11) 〈Rδ,D0,Δ〉=π2 Erf[γδgD0Δ/πg214]γδgD0Δ/πg214 

where $Erf\left(y\right)=\frac{2}{\sqrt{\pi }}\int_{0}^{y}\exp\left(-t^{2}\right)dt,$ is the error function.

**Figure 4:**
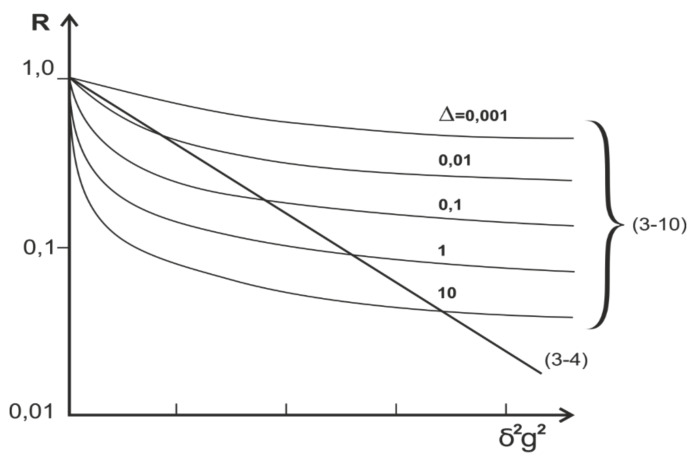
Schematic behavior of R factor from $\left(\delta^{2}g^{2}\right)$ for the case of diffusion in a free volume (straight line (4)) and through narrow pores with variation of diffusion time (10).

Although particle diffusion through narrow pores is not limited, the dependencies of the *R* factor (10) differ significantly from those for unrestricted, free, three-dimensional diffusion of the dependencies of factor *R* (4), as shown in Figure 4.

For yeast cells, the qualitative coincidence of theoretical dependencies is obtained (9) with experimental ones, which allowed authors to affirm [26] the fact that on can observe, via gradient NMR, the single-row motion of water molecules in the pores of yeast membranes. With a lot of questions surrounding this statement, it is necessary first of all to solve the question of the speed of relaxation in narrow pores, which, due to the tightness of the movement, can be so great that it will not allow observing the signal at long diffusion times.

## 4. Restricted Diffusion

When measuring diffusion with gradient NMR, a (non-zero) diffusion time value is required for the accumulation of the phase of the Larmor precession of nuclei, 2τ,Δ, and for this, time proved to be a critical factor in the study of heterogeneous porous systems, leading to the observation of the phenomenon of restricted diffusion. The essence of the phenomenon is that diffusing molecules during diffusion experience impact with the walls of the pores and, as a result, shift to a smaller distance than they could shift in a free, large volume. Experimentally restricted diffusion is manifested in the dependence of the measured self-diffusion coefficient on diffusion time, 2τ,Δ, and this effect has been observed in many heterogeneous systems [4,16,17,23,27,28]. The ratio for R factor ($g_{0},\tau ,D,a$) for the case of diffusion restriction between parallel impermeable planes separated by distance, *a,* is given in Robertson’s work [29] as:

(12) $$R\left(g_{{}^{\circ}},\tau ,D,a\right)=exp\left\{\frac{8a^{2}\gamma^{2}g_{{}^{\circ}}^{2}}{\pi^{6}D^{2}}\cdot \overset{\infty }{\underset{n=0}{\sum }}\frac{1}{{\left(2n+1\right)}^{6}}\cdot \left[1-\frac{3-4exp\left[-\frac{{\left(2n+1\right)}^{2}\pi^{2}D\cdot t}{2a^{2}}\right]+exp\left[-\frac{{\left(2n+1\right)}^{2}\pi^{2}D\cdot t}{a^{2}}\right]}{{\left(2n+1\right)}^{2}}\right]\right\}$$

For $t\ll a^{2}/\pi^{2}D$

$R=exp-\gamma^{2}g_{{}^{\circ}}^{2}D\frac{t^{3}}{12},$ where t=2τ.

That is, expression (12) corresponds to (1) for unrestricted diffusion.

For $t\gg \;2a^{2}/\pi^{2}D,$ expression (12) becomes:

(13) $$R_{\infty }=exp\left[-\frac{\gamma^{2}g_{{}^{\circ}}^{2}a^{4}}{120D}\left(t-\frac{17a^{2}}{56D}\right)\right]$$

It follows from Equation (13) that lnR∝ $g_{0}^{2}$ at all values t, which allows comparing (12) and (1) to formally introduce the coefficient of restricted diffusion:

(14) $$D_{rest}=\frac{a^{4}}{10D}\left(\frac{1}{t^{2}}-\frac{17a^{2}}{56Dt^{3}}\right)$$

At $t\gg \;t_{d}$, where $t_{d}=$ $a^{2}/D$ characterizes the average run time of molecules of distance between barriers, of (14), if we know the D value, we can find a=10D·Drestt214.

**Figure 5:**
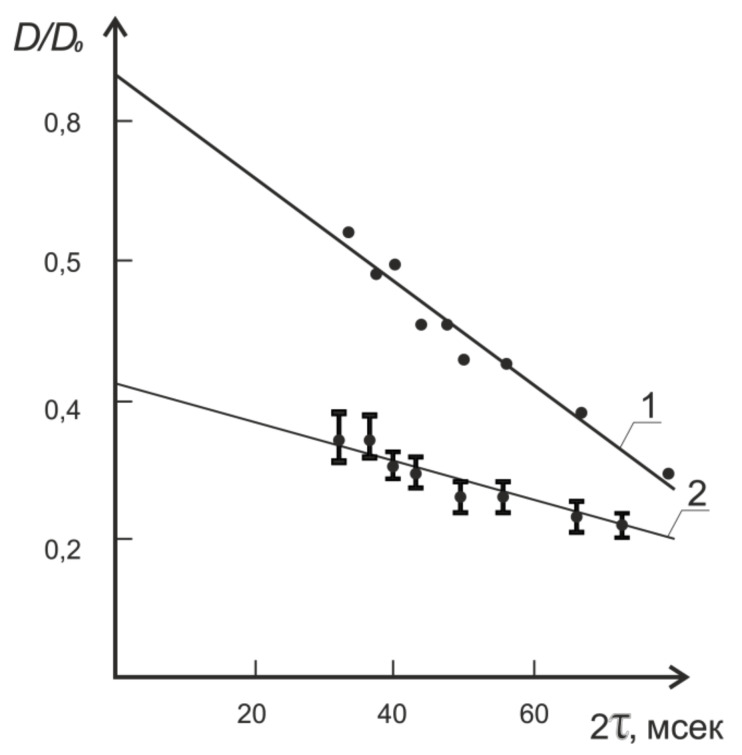
Dependence of relative self-diffusion coefficient on observation time 2τ for Zea mays (1) and for frog liver (2) at 25 °C (Adapted from [4]).

It was the constant gradient method that was originally used in experiments to measure the self-diffusion of water molecules in plant tissues [4]. It is this method that established the phenomenon of the diffusion coefficient reduction with the increase in the experimentally given diffusion time, 2τ, as shown in Figure 5.

Extrapolation of the dependence of the self-diffusion coefficient to a zero value of 2t resulted in the value of the self-diffusion coefficient being close to that for bulk water, which in turn caused the authors to change their position on the water condition in cells, because for the most part, it did not differ from that of free, bulk water [4].

Similar to the problem of restriction of the diffusion of molecules between parallel impermeable planes solved for the constant gradient method, a similar problem is solved for the pulse gradient in obtaining a ratio for *R* [27]:

(15) $$R=\exp\;(-\gamma^{2}\delta^{2}g_{∥}^{2}D\cdot \Delta )\left\{\frac{2[1-\cos\gamma \delta g_{⊥}a]}{{\left(\gamma \delta g_{⊥}a\right)}^{2}}+4{\left(\gamma \delta g_{⊥}a\right)}^{2}\cdot \sum_{n=1}^{\infty }\exp\left(\frac{-n^{2}\pi^{2}D\Delta }{a^{2}}\right)\;\frac{\left[1-{\left(-1\right)}^{n}\cdot \cos\left(\gamma \delta g_{⊥}a\right)\right]}{\left[{\left(\gamma \delta g_{⊥}a\right)}^{2}-{\left(n\pi^{2}\right)}^{2}\right.}\right\}\;$$

where $g_{∥,\;}g_{⊥}$ are components of the gradient vector parallel and perperndicular to the planes, respectively.

Similar ratios are obtained for restrictions in cylinders and spheres [27].

Special features of the ratio (15) are as follows: First, its anisotropy, i.e., dependence on the gradient vector direction relative to the limiting planes. Second, with restricted diffusion, the R factor becomes a function of two variables, $\left(\delta g\right)$ and Δ, whereas with unlimited diffusion, it becomes a function of only one $\delta^{2}g^{2}\left(\Delta -\frac{1}{3}\delta \right).$ Finally, at the orientation of the gradient vector perpendicular to the planes, with an increase in Δ, the echo amplitude tends towards an asymptotic value $R_{\infty ,}^{{}^{\circ}}$ that is independent of Δ, since with Δ= $a^{2}/D\;$, diffusing molecules are locked in the bounding compartment, and a further increase in the diffuse shift of molecules is impossible.

(16) $$\underset{\Delta \to \infty }{\lim}R\equiv R_{\infty }=\frac{2\left[1-\cos\left(\gamma \delta ga\right)\right]}{{\left(\gamma \delta ga\right)}^{2}}$$

(17) limγδg→∞lnR∞≡lnR∞°=γδg2a212 

For the radius sphere ϑ: limγδg→∞lnR∞≡lnR∞°=γδg2ϑ25.

Such *R* behavior is an indicator of restricted diffusion.

The phenomenon of restricted diffusion assumes new meanings for porous objects with permeable walls, which is especially the case for the cells of biological objects.

Due to the permeability of cell membranes, water molecules are not completely limited in their diffusion motion but are only inhibited by the semi-permeability of membrane structures and can theoretically visit all points of sample volume.

One method for analyzing objects with permeability effects is to distract from the details of the microscopic structure of the object. The medium is considered to be homogeneous and they look for an effective diffusion coefficient of this homogeneous medium equal to that in the real sample [30]. On this path in [28], a ratio for unlimited diffusion is proposed to be used to estimate permeability, where the value *D* is replaced by an effective diffusion coefficient—$D_{eff}$:

(18) $$R=exp\left[-\gamma^{2}\delta^{2}g^{2}\left(\Delta -\frac{1}{3}\delta \right)D_{eff}\right]$$

Another similar approach was based on observations that the permeability phenomenon results in additional attenuation of the factor $R_{\infty }^{{}^{\circ}}\left(\delta ga\right)$ characterizing the mode of completely limited diffusion. As a result, in [31], the ability to estimate this additional attenuation by a combination $R_{\infty }^{{}^{\circ}}\left(\delta ga\right)$ with the ratio (18) was realized:

(19) $$R_{\infty }\left(\Delta \right)=R_{\infty }^{{}^{\circ}}\left(\delta ga\right)exp\left[-\gamma^{2}\delta^{2}g^{2}\left(\Delta -\frac{1}{3}\delta \right)D_{eff}\right]$$

The value $D_{eff}$ is defined from the dependence $R_{\infty }\left(\Delta \right)$ at Δ ≫ $a^{2}/D.$

In the systems with a set of relaxation times, when measuring diffusion by a sequence of stimulated echo, the redistribution factor of relaxation times can contribute to a diffusion decline, that is the lack of approaches that use diffusion time, Δ, variation to determine $D_{eff}$.

In a further statement, to separate the cases of impenetrable and permeable pores and the symbols used in the literature for the indication of parameters, diffusion time Δ is remarked by t_d_, i.e., Δ = t_d_ and the experimentally measured value $D_{eff}$ by $D_{s}\left(t_{d}\right)\;,\;т.е.\;D_{eff}$ = $D_{s}\left(t_{d}\right).$

Within the framework of the new notations, the expected idealized dependence $D_{s}\left(t_{d}\right)$ on diffusion time for the case of permeable reservoirs should have the characteristic form of an S-curve.

Characteristic regions of variation *D_s_* from diffusion time *t_d_* allow one to divide the analysis of behavior *D_s_* from *t_d_* to three modes: Short-time translational offset mode, intermediate diffusion time mode, and long-time mode [23].

In the short-time mode (top branch of the S-shaped curve), the translational offset of molecules is much smaller than the size of the cells. The form of diffusional decay is close to exponential, and the diffusion coefficient is constant and close to the free-water diffusion coefficient *D_o_*. The diminishing middle part of the S-shaped curve responds to the region with restricted diffusion, where there is no averaging of local translational movements of water over a sufficiently large volume, *D_s_*, whereby it feels for the obstacles in this mode and decreases with the growth of *t_d_*. Finally, the lower branch of the curve, owing to the area of hindered diffusion, depends on the process of intercellular permeability, which provides the ability to achieve the effect of averaging the diffusion at distances exceeding the size of pores (cells). In this area, the water diffusion coefficient *D_∞_* is independent of *t_d_* and less than *D_o_*.

To estimate the actual permeability coefficient in [28] from *D_s_*, the ratio obtained from the analysis of resistance to the translational displacement of molecules of the series of plane-parallel membranes by permeability *P*, separated by distance, *a*, is usually used [32]:

(20) $$\frac{1}{D_{\infty }}=\frac{1\;}{D_{0}}+\frac{1}{Pa}$$

In plant tissue, intercellular transport is carried out through parallel pathways: Through the plasmalemma with the release of water molecules into the extracellular space, and through intercellular channels—plasmodesmata [6,7,33]. In ratio (20), there is no binding to the details of intercellular transfer mechanisms. This fact allows it to be used to evaluate the total effective intercellular permeability *P_eff_* of the membrane + plasmodesmata complex. The characteristic limitation size required for computation, *a*, which is usually associated with the size of cells, can be determined from the well-known Einstein–Smolukhovsky ratio:

(21) $$D_{s}\left(t_{d}\right)=\frac{a^{2}}{6t_{d}}$$

**Figure 6:**
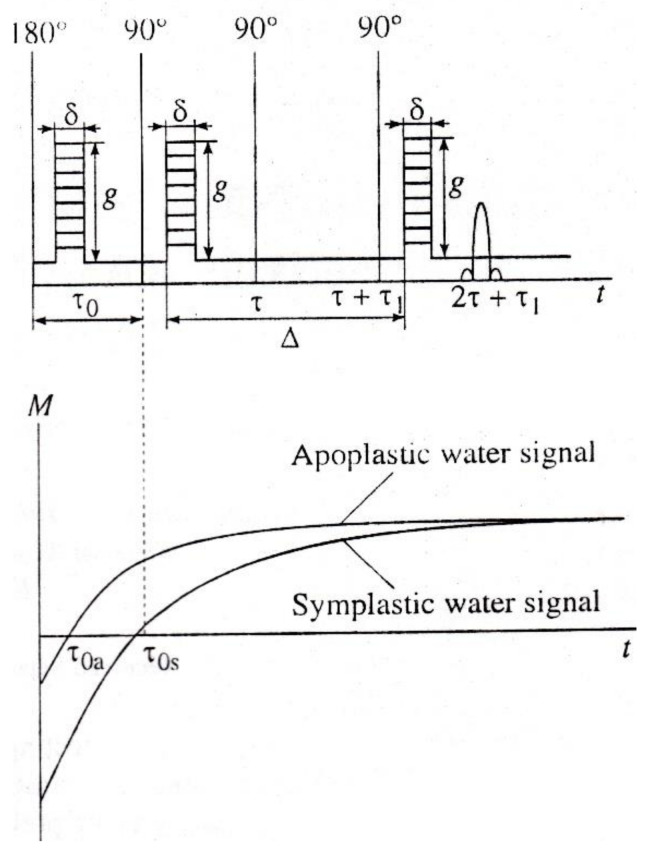
Tandem inclusion of pulse sequences of measurement of relaxation (zero-method) and diffusion (adapted from [51]).

The value of the diffusion coefficient $D_{s}\left(t_{d}\right)$ shall be recorded under the conditions of complete diffusion limitation, when the measured diffusion coefficient becomes a linear function from the inverse diffusion time (*t_d_*^−1^) [23,34]. However, with the permeability of restricted barriers, this dependence is distorted (Figure 6) and, as a result, the problem of eliminating the effect of permeability on the dependence occurs $D_{s}\left(t_{d}\right)$. This problem was solved by the renormalization of the $D_{s}\left(t_{d}\right)\;$dependency to the dependency *D_ef_*(*t_d_*^−1^) (scaling approach) [35,36]. Renormalization for the area of long times *t_d_* is set by the ratio [35]:

(22) $$D_{ef1}=D_{0}\frac{D\left(t_{d}\right)-D_{\infty }}{D_{0}-D_{\infty }}\;$$

Due to the limitation of *t_d_* by relaxation time, the value *D_∞_* often cannot be directly defined, and then renorming *D_∞_* is positioned as a fitting parameter. It is worth noting that the number of works on the theory and practice of applying gradient NMR to the study of porous media with random obstacles is increasing steadily, which indicates the potential of gradient NMR [23,34,35,36,37,38,39]. Among the most complete theories of diffusion attenuation of the spin echo signal in media with random obstacles is the work of Fatkullin [37]. An analogous theory was created by Mitra and Sen two years later [38]. For plant objects, the possibility of estimating the permeability factor of membranes by the procedure for determining all necessary parameters within a single gradient NMR experiment places it in the category of indispensable methods in plant physiology.

## 5. Stages of Development of Gradient NMR Technique

The technique of a constant gradient of low magnitude for measuring the diffusion of the order of 10^−15^ m^2^/s is simple enough in its schematic solutions and lies in the transmission of direct current (usually up to 1 A) through special gradient coils located in the NMR sensor. The main focus of developers is on the design of gradient coils, which must create a linear gradient in the volume of the study sample, be unleashed electromagnetically from transceiver circuits of the NMR sensor, and be structurally coordinated with other elements of the sensor, including the thermostatic channels of the sample. Anderson planar coils are most commonly used as coils [40].

The pulse gradient technique is much more difficult to implement, due to the need to provide the identity of the area of gradient pulses with relative instability of no more than 10^−4^ at currents through gradient coils in tens of amperes. In the first developments, saturated transistor keys powered by batteries were used [41]. Then, two-channel circuits powered by AC appeared, in which one pulse was a reference with a discretely variable amplitude, and the second was tuned to the first. An indicator of the equality of pulses was the formation of the echo maximum at time 2t [42].

With the advent of available high-quality operating amplifiers, pulse generation schemes with stabilization of pulse area by means of feedbacks have spread [43,44]. previous authors [44,45] published a very successful circuit of the precision stabilizer with an integrated value of current pulses for an inductive load and a sensor design with gradient coils, which was developed by the staff of Kazan Federal University. It allowed one to generate gradient pulses up to 50 T/m at a current of about 100 A, with relative instability of integral values not exceeding 10^−6^. The use of this scheme made it possible to use the technique of automatic recording of diffusion recessions with accumulation and to measure diffusion coefficients up to 10^−15^ m^2^/s. Later, the same team obtained gradients with an amplitude above 200 T/m.

In pulsed gradient NMR devices, the quadrupoles sinus coils have taken root as gradient coils, providing satisfactory linearity and magnitude of the gradient per current unit [46].

Attention in development has been paid to the nuances of the application of gradient NMR in the mode of short diffusion times. This mode is characterized by a dense arrangement of RF and gradient pulses and, as a result, a negative influence on the results of measurements of gradient pulse fronts. The introduction of additional gradient pulses of front compensation and the preparation of the configuration of the magnetic field into standard pulse sequences [47] made it possible to reduce the influence of the fronts.

## 6. Methodological Approaches to Selective Measurements of Diffusion

Common spin-echo NMR sequences with pulse gradients of the magnetic field used in studies of aqueous transport in plants work as “brutto-methods”, with the contribution of magnetization from all water fractions. Since parallel pathways of transfer—apoplast and symplast—simultaneously operate in plant tissues, it is advisable to develop simple methodological approaches to selective registration of diffusion through one transfer pathway or another. Selectivity is realized by suppressing the contribution of magnetization signals from water components of no interest to the measured echo signal. Suppression is provided by either a diffusion or relaxation mechanism, including paramagnetic doping. Examples of such selective techniques are as follows: (1) The method of paramagnetic doping with relaxation suppression of the signal of magnetization from the water of the extracellular space by paramagnetism [48]; (2) a tandem mix of diffusion and relaxation sequences in which the diffusion sequence suppresses the signal from rapidly diffusing aqueous components; (3) a tandem inclusion of pulse sequences of relaxation measurement (zero-method) and diffusion [49]. The inclusion of the diffusion program of pulses at the time of the transition of magnetization through zero, as shown in Figure 6, gives the possibility of selective suppression of the contribution to the total magnetization signal from the water component with long spin-lattice relaxation time T_1_ (from symplast water, as a rule).

Suppression of the interfering signal from the vascular system water is realized with the addition of two pulses of the magnetic field gradient directed through the current and included before the regular gradient pulses with a gradient vector directed transversely to the plant. For such sequences, it is advisable to use a two-circuit gradient pulse generation scheme [50], which uses two amplifiers and two sets of gradient coils, for gradients in Z and Y directions, respectively. When registering echo signals, a phase-sensitive detector must be used.

The program presented in Figure 6 was used for selective assessment of water transport through apoplast of corn root [51] and cytoplasm of Elodea cells [52,53].

It is worth noting that the greatest success in the development of the theory and technique of gradient NMR for the study of diffusion in porous systems and in heterogeneous systems in general was achieved at the Institute of Physics of Kazan Federal University (Russia) [23,54].

## 7. Special Features of Experimental Studies of Biological Objects

The main feature of studies on biological objects is related to the processes of continuous metabolic changes. Unlike non-living physical objects, biological objects are continuously changing because of the processes of metabolism during the time of the experiment and, especially, from the moment of preparation. This fact leads to special experiments to confirm the preservation of functionality close to the clock in the intact state using the comparative method of experience/control analysis, with regard to the representative of the sample for average biological spread and repeated measurements (repetitions). Nevertheless, a priori fact of simultaneous flow in samples of a number of interrelated processes leads to the need for “smoothing” interpretation by deliberate exclusion of details that, according to the researcher, do not noticeably affect the main characteristics studied. This is also facilitated by the fact that many metabolic processes only have qualitative representations and, moreover, some biological tasks do not require extensive control of all, even quite known, factors. The comparative method of experience/control analysis is widely used in plant physiology and allows one to greatly simplify the translation of experimental data from the language of NMR parameters into the language of physiologists, often without going into detail of the physics of the process [5]. As a result, the parameterization procedure and terminology of effective parameters are often used in the analysis of experimental data. The parameterization procedure often consists of describing experimental data by known functional dependencies, often exponents or the sum of exponents, even if more qualitatively complex behavior is known. Such cases include the formal separation of DD into components of restricted diffusion and unrestricted diffusion over the chosen time, and this makes sense since the restricted component is more sensitive to permeability. For example, the case may be that gradient NMR examines the effect of membrane-active compounds on diffusion and it is obvious that diffusion motion is simultaneously controlled by the phenomenon of restricted diffusion, the complicated distribution of the size of compartments, their permeability, the contribution from water flows through parallel transport routes through the symplast and extracellular space, and the exchange of free and bound water. Furthermore, if the diffusion decay of magnetization signals within the corridor of typical dispersion of experimental values is satisfactorily described by the exponent, then the concept of the effective diffusion coefficient is introduced, where under the sign of Deff efficiency, either obscure or not essential in the moment, the details of the transfer processes are hidden. In this sense, the coefficient of self-diffusion in biological objects in most cases initially carries the meaning of an effective diffusion coefficient. To refer to this approach, the term “formalism of effective diffusion coefficient” is used.

It is obvious that this concept is more capacious than the Deff value introduced for the average analysis of microstructure details of porous media (see Section 3 and Section 4).

## 8. Gradient NMR in the Studies of Diffusion Transfer of Water through Different Pathways in the Root of Plants

Special features of water transfer through different pathways in plant tissues, such as the switching of pathways in changes in external conditions, are an important problem for plant physiologists. Challenging tasks include the research of symplast transport through plasmodesmata. Plasmodesmata cannot be dissected in its pure form, and gradient NMR proved to be almost the only non-invasive method that suits the study of transport through plasmodesmata.

### 8.1. Transfer of Water through Cytoplasmic Symplast through Plasmodesmata

The identification of the structure and functions of intercellular contacts—plasmodesmata—including in terms of water transfer through the symplast system through plasmodesmata, remains one of the central problems of cytology [33]. The relatively recently proposed model of the transport of water at the root of MESNA [55] indicates that the absence of data on characteristic parameters of transport via plasmadesmata is a stumbling block to further refinement of the model.

**Figure 7:**
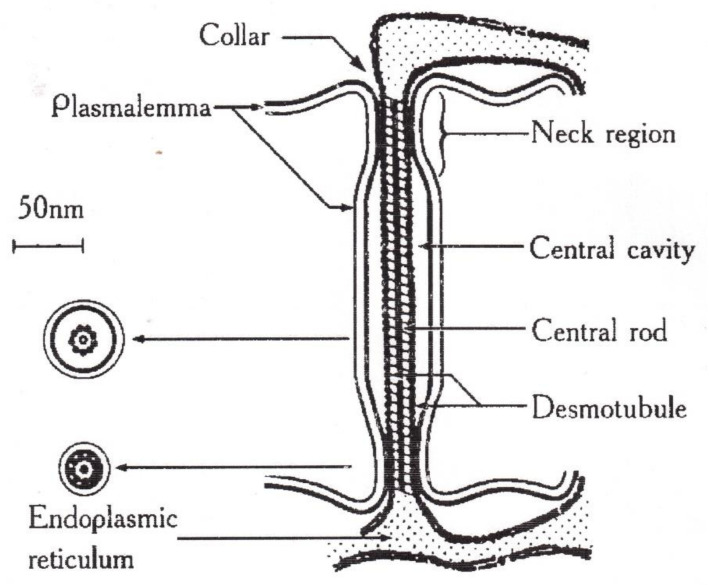
Schematic image of a simple plasmodesma. ([57]).

In the known models of plasmodesmata by Robards [56] and Lopez-Saenz [57], the set of structural elements of plasmodesmata is equal, and the difference is in the interpretation of the structure details as shown in Figure 7:

According to Robards [56], the desmotubule is a hollow tube, and transport is carried out through both a cytoplasmic ring–annulus and a desmotubule. According to Lopez-Saenz [57], the desmotubule is a continuous protein rod, and transfer occurs only by the annulus.

In [58], a number of issues of the plasmodesmata problem are solved within the framework of ideas about the phenomenon of capillary osmosis. The idea is that the phenomenon of capillary osmosis comes from the fact that the plasmodesmata are essentially pore capillaries with a size suitable for manifestation of the effect of capillary osmosis, Figure 7. The theory of capillary osmosis is based on the thermodynamic effect of the influence of the pore wall on solution components [59]. Solution–wall interaction causes radial changes in the solution concentration and accompanying pressure gradients perpendicular to the wall.

**Figure 8:**
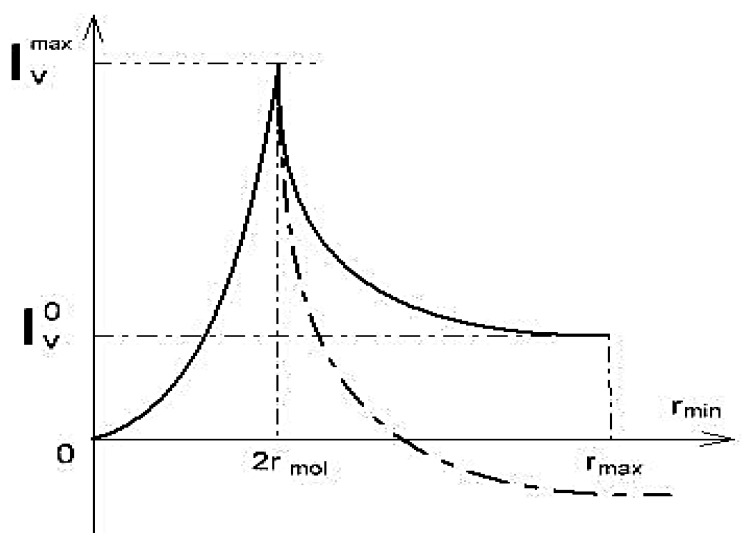
Dependence of water flow I_v_ through plasmodesma depending on the opening of the cervical constriction from the position of full coverage of the desmotubule, *r_min_* = 0 to full opening, *r_max_*. The dotted line corresponds to the situation of the adsorption potential of plasmodesmata walls [58].

Three-dimensional changes in the concentration of the solution inside the capillary cause the driving force of the osmotic flow. Calculations for the variant of the steric interaction of water and osmotic sucrose with plasmodesmata walls led to a functional dependence of water flow through plasmodesmata from the aperture of cervical constriction in plasmodesmata, with an acute maximum at different values of parameters characterizing the geometry of plasmodesmata [58]:

(23) $$I_{V}^{max}=I_{V}^{0}2a/3\left(a^{3}+\frac{3}{4}\varepsilon \sqrt{a}\right)\approx 8I_{V}^{0}\sqrt{a}/9\varepsilon \;\;\;\;\;\;\;\;\;\;\;\;\;\;\;\;=4\sqrt{a}r_{max}r_{mol}^{2}\Delta \pi /9\eta \varepsilon l\;$$

(24) $$I_{V}^{0}\approx k_{0}\frac{\Delta \pi }{al}=\frac{\rho \;r_{max}\;r_{mol}^{2}\;\Delta \pi }{2\eta l}$$

where $I_{V}^{max}$ is the mass water flow at the maximum point, as shown in Figure 8, *a* = 2$r_{mol}^{2}$/ r, *r_mol_*— is the radius of osmotic molecule (sucrose), l is the plasmodesma length, Δπ is the difference of osmotic pressure at the ends of the plasmodesma, η is the water viscosity, ρ is the solution density, $r\left(x\right)$ is the plasmodesma opening, $r_{m,}\;r_{max,}\varepsilon$ are characteristics of the function $r\left(x\right),$ and $I_{V}^{0}$ is the water flow in steady conditions. In narrowing, $r\left(x\right)$ continuously varies from $r_{min}$ to $r_{max}$ (Figure 8): At $r\left(x\right)$ = *r_min_*, the constriction is completely closed and at $r\left(x\right)$ = $r_{max}$ it is completely open. The introduction to the task of the adsorption interaction of osmotics instead of the steric interaction through the walls of plasmodesmata showed that changes in the aperture of the crevice narrowing can not only affect the value of mass flow, but also the direction of the flow (dotted in Figure 8). With an open aperture, the flows of solvent and solute are unidirectional. When the aperture increases, the flow of the solvent (water) changes direction to the opposite (Figure 8. dotted). The obtained results support the notion that plasmodesmata is not just a transfer channel, but also an acutely controlled valve system. In [60], using the example of Nicotiana clevelandii trichoma with the use of dyes, the occurrence of an irreversible blockage of plasmodesmata at a pressure gradient between cells of 200 kPa is shown. It may be due to the reaction to the prevention of water loss during depressurization of water transfer channels of plants.

**Figure 9:**
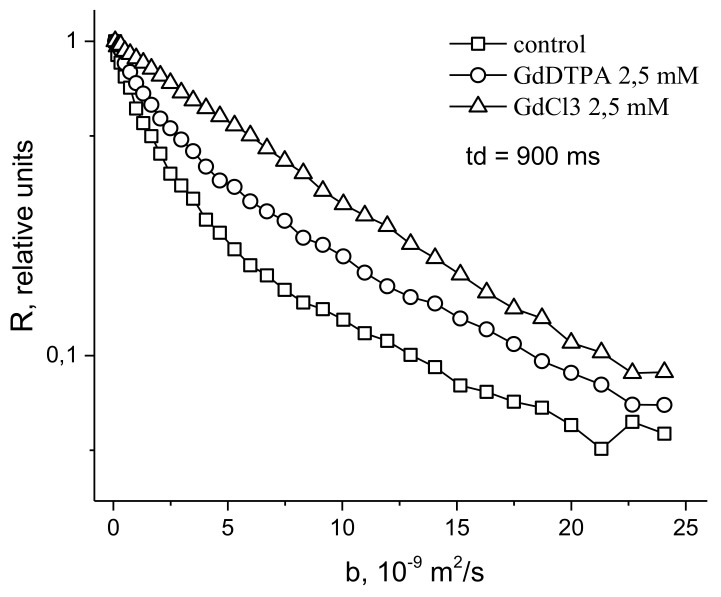
Typical dependence *R* (b ($\;b=\gamma^{2}\delta^{2}g^{2}t_{d}$) for root segments normally and under the influence of Gd DTPA and GdCl_3_.

In [48], in order to study the symplast transfer, it was proposed, before diffusion measurements, to use pre-introduction into the extracellular space of the root tissue of paramagnetic ions of a high relaxation efficacy (paramagnetic doping), which does not penetrate the cells and plasmodesmata, accordingly. Paramagnetic ions (PMI), dramatically accelerating the process of magnetic relaxation of extracellular water, allow one to exclude the contribution from the magnetization of water of the intercellular space in the recorded diffusion decay. As a result, the observed visible diffusion run is localized to the limit of cell volume because when the water molecule leaves the cell in terms of NMR, signal observation becomes invisible due to the rapid relaxation on PMI. If the cells are bound by water channels (plasmodemata), the observed diffusion run will exceed cell sizes, which is a signal for water transfer through intercellular channels. Among the available paramagnetics of high relaxation efficiency, MnCl_2_ is suitable at a concentration of 0.02–0.03 M [61], at which T_1,_ T_2_ of water is about 10–15 ms and 1, 4–2 ms, respectively. However, the plasma membrane is quite permeable to manganese ions [61], so the researchers’ attention shifted to the use of rare-earth gadolinium ions, which also have a high relaxation efficiency, but do not penetrate cells. Moreover, for NMR tomography based on gadolinium and manganese, contrasting complexes with an “attachment” are synthesized, Gd DTPA and Mn DCPA, which do not penetrate cells and are convenient for use in the research of symplastic transfer [62,63,64], as can bee seen in Figure 9:

The control is characterized by the non-exponential form of *R* (*b*), which approaches the exponential form with the paramagnetic exception of the contribution to the signal from the rapidly diffusing fraction of extracellular water.

Experimental results of the gradient NMR, which demonstrate the transfer of water through the root symplast, gave a new look at the problem of long-distance transport in the compromised model, where part of the flow is provided by the vascular system and the other part is provided by symplast.

**Figure 10:**
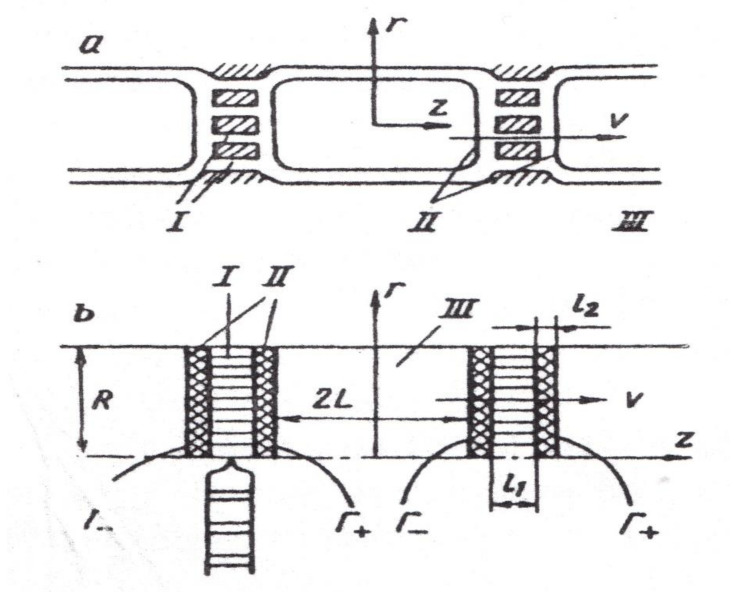
Schematic representation of the cell chain divided into zones: I—plasmodesmata; II—tonoplast; III—vacuole [65].

In [65], the theoretical description of the transfer through the symplast of a linear chain of cells with the establishment of a connection between the experimental coefficient of water diffusion and the linear transfer rate *V*_0_ is given. To find the connection between *v*_0_ and *D*, a hydrodynamic model of water run through a linear chain of cells formed by periodic alternation of zones is considered, shown in Figure 10: I—plasmodesmata; II—tonoplast membranes; III—vacuoles.

In the case of piston flow mode, the task allows for an explicit solution:

(25) $$\frac{D_{э\phi }}{D}=1-\frac{1}{2P_{e}}\frac{\left(exp2P_{e}-1\right)\left(expP_{e}b-1\right)}{expP_{e}\left(2+b\right)-1}\;$$

where $P_{e}=\frac{V_{o}L}{D}$ is the Palk diffusion criterion, constructed for the cell, $b=l_{1}/mL,$ *m* is the porosity of zone -I, *D* is the coefficient of diffusion of free water, along with the other parameters (see Figure 10).

For laminar flow, the task is solved numerically. For the specific case of corn root at *D_eff_*_._ = 1.7 × 10^−5^, *m* = 0.01, *L* = 40 mkm, *l*_1_ = 0.5 mkm, *D* = 2.5 × 10^−5^ cm^2^/s, *V*_0_ is rated as 7 × 10 cm/sec for piston and 6 × 10 cm\s for laminar flow. The obtained values are close in magnitude and, on average, two or three orders of magnitude below the xylem flow rate (0.3–1.6 cm/s), according to [66].

### 8.2. Controversial Model of Vacuolar Symplast Transport

The problem of water transport through plasmodesmata received a new surge of attention due to the development of ideas about a single endoplasmic continuum of the plant organism [67,68] and the new opportunity to assess the transport of water through symplast by gradient NMR in tandem with paramagnetic doping.

According to the existing ideas, since ancient times, vacuoles have represented closed, restricted membrane–tonoplast vesicles and are, as a rule, a component of mature plant cells. The proven role of vacuoles is to maintain turgor pressure and deposition of excess metabolites. With the development of new views about the single endoplasmic continuum of the plant organism united by transport channels of plasmodesmata, these representations have been revised [67,68,69,70].

**Figure 11:**
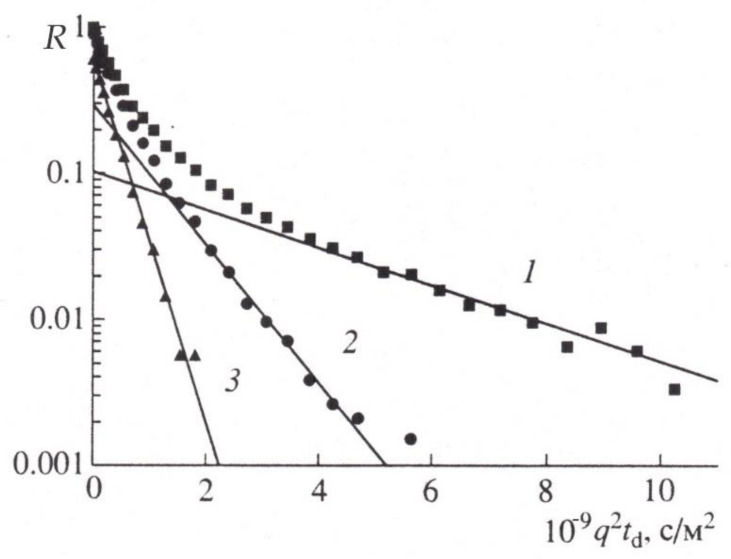
Diffusion decay of echo in wheat roots in the normal conditions (decomposition into three exponents, (*q*^2^ *t_d_* =$\;\gamma^{2}\delta^{2}g^{2}t_{d\;},$ *t_d_* = 100 mc) [71].

**Figure 12:**
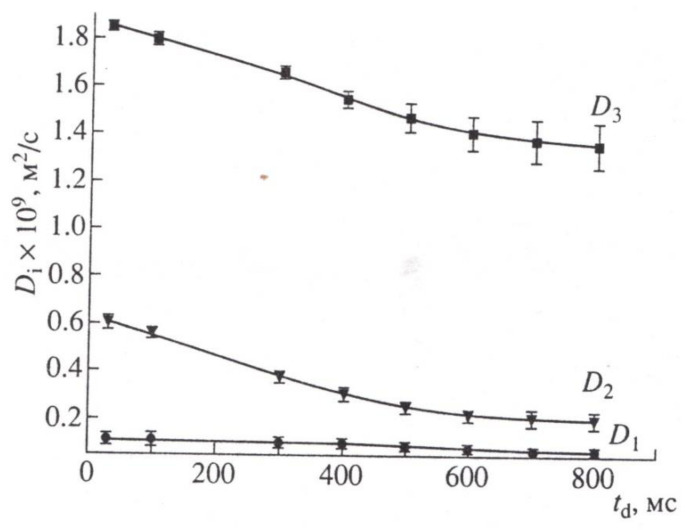
Dependencies *D*_1_, *D*_2_, *D*_3_ on diffusion time if there are manganese paramagnetic ions in root apoplast (MnCl_2_, 10 mМ, 15 min) [71].

To experimentally substantiate the new representations in [70] with the example of wheat roots, the behavior of magnetization in the range of three orders of magnitude for a sample of roots in normal and under paramagnetic doping is analyzed. It was demonstrated that the diffusion decay of magnetization of water in the root of wheat can be represented by the sum of three components, as shown in Figure 11:

When the signal from the apoplast water was excluded by paramagnetic doping (MnCl2, 10 mM), three components with characteristic features of water transfer in porous media remained observable. One component had high diffusion mobility, which allowed the authors to attribute it to the transfer of water through vacuoles, which have no effective barriers to diffusion [71], as shown in Figure 12.

**Figure 13:**
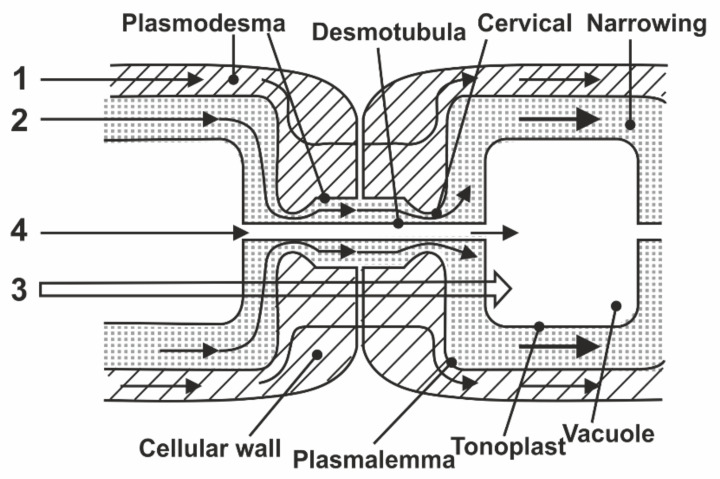
Model of transfer by a vacuolar symplast through desmotubule: (1) Apoplasmic way through the cells walls; (2) symplast way on cytoplasm plasmodesmata; (3) cell-to-cell way through tonoplast, cytoplasma, plasmalemma, and cell walls; (4) symplast vacuolar way through desmotubula plasmadesmata and vacuole.

In accordance with Figure 13, the correlation of coefficients with the feasible water fractions in the root is as follows: *D*_1_ characterizes the slowest fraction bonding with part hydrate water, water enclosed in cell vesicles, and water diffusing with them; *D*_2_ characterizes the average of the mobility fraction of the cytoplasmic symplast water, diffusing in cytoplasmic ring (annulus) of plasmadesmata; and *D*_3_ characterizes the self-diffusion of water in the vacuolar symplast system.

The results obtained in total allowed the authors [70] to divide the previous definition of symplast as a single system into two subsystems: vacuolar and cytoplasmic symplast. In this model, symplastic transfer through plasmodesmata is carried out both through the cytoplasmic ring with cervical constrictions and through desmotubules connecting vacuoles, as shown in Figure 13:

Figure 13 shows the connection between the vacuoles via the desmotubule of the plasmodesmata.

Based on the experiments with external influences—osmotic stress, physiologically active compounds (pipolfen), and inhibitors of actomyosin sphincters of plasmadesmata (cytocholasin B)—in experiments with plants with different resistance to water stress, high reactivity of changes in coefficients was established, D_2_, D_3_, and in some cases in counterphase. According to the authors, the counterphase is explained by the increase in the diameter of the desmotubule with a corresponding decrease in the open area of the cytoplasmic ring and vice versa [70,71,72,73,74].

The experimental data presented offer a great deal of food for thought, but it is important to note that not all physiologists are open to the suggested ideas about vacuolar symplast. In [75], the opponent gives at least five arguments to consider that by now there is scant evidence in favor of the reality of the vacuolar symplast and the data [70,71,72,73,74] require other and more cautious interpretation.

### 8.3. Apoplast Transfer

As is known, the volume of water in apoplast of root tissues is about 10–15% of the total water content, and therefore in NMR measurements, the signal from apopalst water is masked by a powerful signal from the symplast water, first of all, in vacuoles. The problem of selecting a relatively weak signal from apoplast water becomes simpler if a selective approach is used.

In (51), the diffusion over the apoplast was recorded by gradient NMR under the conditions of excluding the contribution to the signal from vacuoles water. For that purpose, the sequence of stimulated echo with gradient pulses was included after a preliminary inventory 180° RF pulse (see Figure 6). The moment of inclusion was chosen when the magnetization from vacuole water passed through zero (see Figure 6). With the help of selective measurements, it was possible to obtain evidence of accelerated diffusion of water in the axial direction of the root (variation of the relative diffusion coefficient in the range of 1–1.5). It is assumed that the apoplastic flow balances the inversely directed active transfer of water through the cytoplasmic symplast (51). Possible multidirectionality of flows is due to mechanisms of the dynamic mode of maintaining the level of water content of the root in the range of regulation of water absorption. Multidirectionality does not seem too unexpected. This theoretically predicts the possibility of variation of pressure in apoplast from negative to positive. Given the representations of direct connection of the apoplast volume with the external environment, pressure variations can only be realized in the dynamic mode with a noticeable resistance to transfer at the inlet/outlet of water from apoplastic space [76].

### 8.4. Transmembrane Water Transfer through Aquaporins in the Studies of Aquaporin Permeability of Membranes

According to modern ideas, transmembrane water-conducting protein channels, aquaporins [77,78], play a primary role in the intercellular transfer of water in a transcellular way.

It is believed that these channels provide at least 70% of intercellular transmembrane water transfer [77], and more than three dozen types of aquaporins have been identified in maize [78,79]. It is shown that aquaporin activity can be suppressed by mercury chloride HgCl_2_, which is indicated as an effective aquaporin blocker [80,81]. Mercury ions are believed to interact with free sulfhydryl groups of proteins that form water channels and through conformational changes, cause their closure.

In [82], the method of gradient NMR was used to investigate diffusion transfer of water in the radial direction of the roots of corn sprouts (*Zea mays* L.). in normal conditions under the influence of 0.1 mМ HgCl_2._

The established fact of reducing diffusion water transfer from the norm by 1.5 to 2 times under the influence of HgCl2 was manifested only on samples grown in solution with Ca2+ (Ca^2^+ion concentration of 1.5 mm) and could be reversed using β-mercaptoethanol. The latter indicates that the reduction of transport is obligated to block aquaporins.

The possibilities of gradient NMR in the dimension of diffusion transfer with steps from one second and higher are realized in the experiment with one-time feeding of osmotic stress to the roots [83]. In order to obtain information about the connection of different root zones in response to osmotic stress and simultaneously avoid problems with the suppressing signal of magnetization from the osmotic solution, the osmotic stress (PEG-6000) was created in the meristematic zone of the root, and diffusion was measured in the zone of the root 5 cm above the incubation zone in osmotics. Based on the idea that different cellular compartments have their own relaxation times [84,85], a quantitative assessment of the diffusion transport of water in corn roots under the action of PEG-induced water stress was carried out on the basis of the analysis of diffusion decays (DD) of spin echoes measured at different diffusion times *t_d_* = 15, 100, and 700 ms. It was believed that at *t_d_* = 15 ms, the signal from the bulk of extra- and intracellular water is recorded, at *t_d_* = 100 ms the main contribution to DD is made by intracellular water, and at *t_d_* = 700 ms it is mainly the water of vacuoles that is measured.

**Figure 14:**
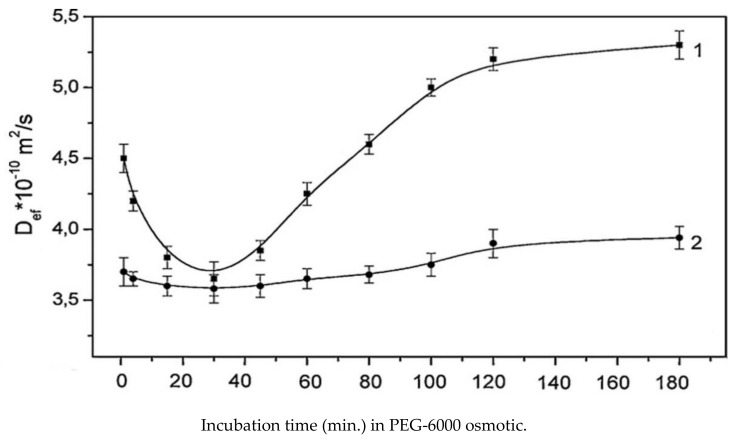
Def dependencies on time (in minutes) of PEG-6000 osmotic action for roots in normal conditions (1) and (2) roots pre-treated with aquaporin blocker (15 min, 0.1 Mm HgCl_2_ at *t_d_* =100 mc), [80].

Diffusion measurements at a diffusion time of 100 ms showed an extreme Deff dependence on the time of osmotic action, as shown in Figure 14, [83].

At the same time, for the sample pre-treated with a mercury blocker, the extreme point is practically not expressed and a relatively stable decreased level of D_eff_ is recorded with a value close to the control D_eff_ value at the extreme point. According to the authors, the extreme D_eff_ dependence clearly demonstrates the sequential regulatory response of aquaporin conductance to osmotic stress that develops over time: The phase of closing aquaporins within 20 min, with the minimum phase at 30 min, and subsequent opening in 90 min with the restoration of the transfer. From the analysis of the D_eff_ dynamics at different times of diffusion, in the comparison experiment with (HgCL_2_)/control, a conclusion was made about the manifestation of multidirectional changes in the permeability of the plasmalemma and tonoplast, associated with down- and upregulation of aquaporins (the PIPs and the TIPs, correspondingly) [83].

**Figure 15:**
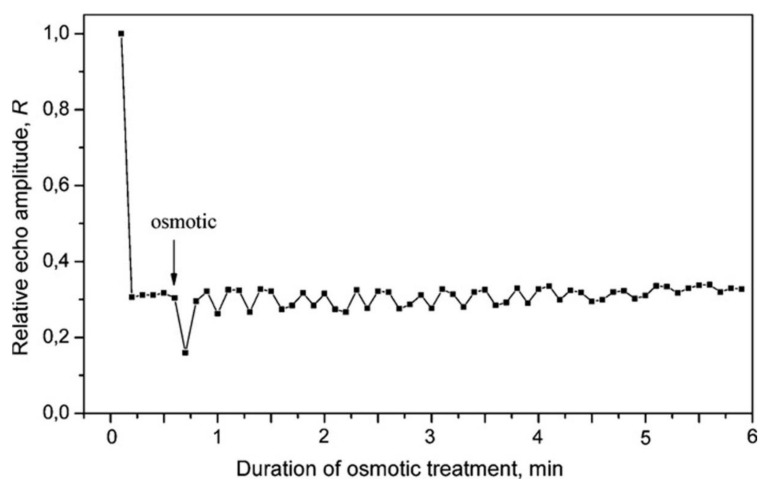
The dip in the curve decay of magnetization near 0.8 min after a fast (pulse) supply of the PEG-6000 osmotic to the meristem of the root, ($\delta^{2}g^{2}t_{d}=const)$ [80].

Interesting data are obtained in [83] on the second dynamics of transfer in response to the PEG-6000 pulse of induced water stress. After rapid flooding of the root meristems with osmosis 1 cm long in the NMR measurement zone of the plant, 5 cm up at a diffusion, time of 15 ms, and with the selected constant parameters of the gradient pulses in the second range, a splash of magnetization decay was recorded, followed by a decaying oscillatory mode within 6 min, as shown in Figure 15.

The dip in the curve decay of magnetization near 0.8 min and the subsequent decaying oscillatory mode, as shown in Figure 15, are associated with the propagation through the root of the hydraulic signal accompanied by fast, spaced in time, multidirectional changes in the permeability of the plasmalemma and tonoplast [80].

Gradient NMR was used to estimate the reaction of aquaporins’ conductivity to the effect of temperature [86]. It is known that the energy values for the activation of water diffusion of Ea through a lipid membrane bilayer is about Ea = 45–60 kJ mol, which is significantly higher than in plant membranes, which is about 17–25 kJ mol [87,88]. Inhibition of water transport through aquaporins by mercury agents reacting with sulfhydryl groups of channel proteins, leading to channel closure, increases Ea to a level characteristic of transport through the lipid bilayer [87].

This fact was used in [86] to assess the contribution of temperature dependence of water transfer through aquaporins to the total conductivity of maize root membranes. The results of temperature measurements show that water transfer through aquaporins is temperature sensitive [86]. In different temperature intervals, the contribution of parallel membrane water transfer pathways (through aquaporins + through lipid bilayer) changes. In the range of optimum temperatures (17–22 °C), the preferred transfer of water is through aquaporins. At higher temperatures (above 25 °C) the contribution of transmembrane water transfer through lipid bilayer increases. However, in the temperature dependence of transfer through aquaporins, the contribution from cytoplasmic flows caused by the work of the actomyosin motor is not excluded. In [52], when gradient NMR selectively records the water diffusion parameters over the cytoplasm of Elodea stem cells, it was shown that the phenomenon of cytoplasmic flow, which is provided by proteins of the actomyosin motor, is superimposed on the intracellular diffusion transfer [89]. As a result, the total translational displacement of water molecules significantly exceeds the displacement due to the self-diffusion of water itself. The actomyosin motor can be activated by an increase in temperature or suppressed by a specific inhibitor of the ATPase activity of myosin, 2,3-butanedione monoxime [90].

**Figure 16:**
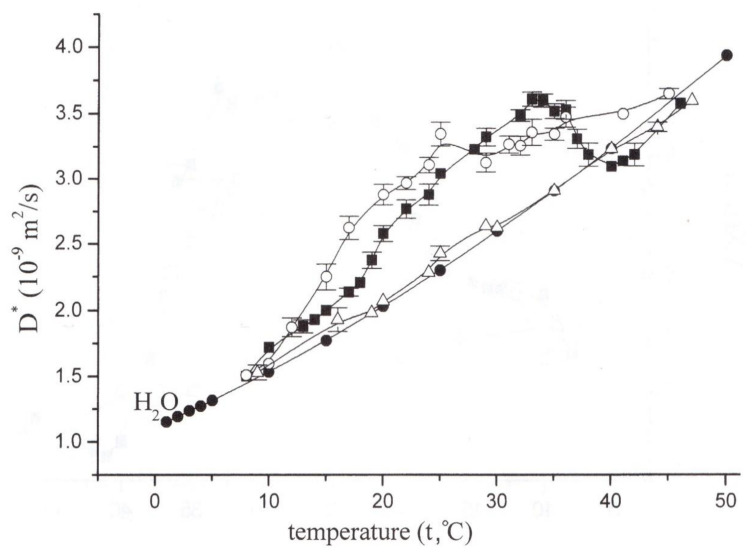
Temperature dependence of the diffusion coefficient of cytoplasmic water in the control (■) and under the effect of gadolinium (○), BDM (Δ), and of bulk water (●) [53].

**Figure 17:**
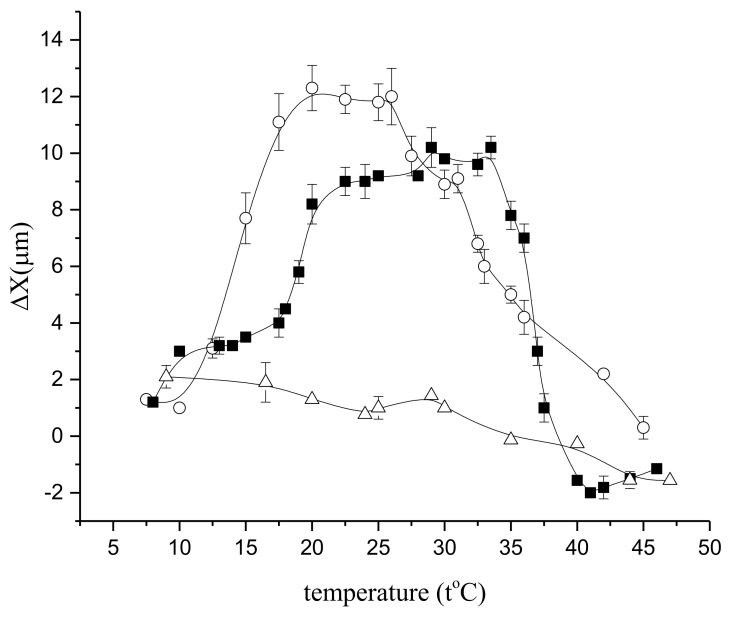
Temperature dependence of Δx in the control (■) and under the effect of gadolinium (○), BDM (Δ) [53].

In fact, in normal conditions, the temperature dependence of the relative diffusion coefficient demonstrates an extreme increase at temperatures from 22 °C, as shown in Figure 16, but under the influence of butanedione monoxime, it falls to a level close to that for free water, and at 40 °C and above it falls below the free water level [53]. The results clearly demonstrate a significant contribution of the actomyosin motor to intracellular water transfer, as well as its conspicuous thermodependence, as shown in Figure 16:

To visualize the contribution of the actomyosin motor, the difference between the diffusion displacement of water molecules in cells and free water was estimated in [53]. Diffusion translational displacement of molecules, X, was determined from the measured values of the diffusion coefficient using the Einstein–Smolukhovsky ratio: X^2^ = 2Dt_d_ at the same temperatures for the same time of diffusion, as shown in Figure 17:

If the diffusion displacement is taken as the reference point (∆X = 0) in the norm, at 30–35 °C, then under the influence of butanedione monoxime at temperatures around and above 30–35 °C, the value of ∆x becomes negative, as shown in Figure 17. It then follows that butanedione monoxime at elevated temperatures not only inhibits the operation of the motor, but apparently destroys actin filaments, with a concomitant increase in the viscosity of the cytoplasm, which is reflected in the value of diffusion displacement.

## 9. Gradient NMR in the Studies of Water Transport in Root Segments and Roots of Intact Plants When the Concentration of the Main Air Components Changes

### 9.1. Growth and Transfer of Water in the Root Segment

Pressure, as well as temperature, are integral factors in the external environment. The functional role of pressure, first of all, is considered from the position of the driving force of mass transfer of aqueous solutions. An analysis of the response of the hydrodynamic system of a plant to the action of external pressure appears to be a promising method for obtaining an answer to the question, can the pressure in the hydrosystem of a plant perform the functions of a regulator of water transport? Gradient NMR studies of the transfer directly under external pressure involve measurements on plant tissue segments placed in a high-pressure ampoule [91]. In turn, the use of segments has a set of features associated with the obvious fact of interruption of the xylem and phloem channels for the transport of aqueous solutions and the release of pressure in the plant’s hydraulic system, which can reach 5–6 bar [92,93]. It therefore follows that, along with studies of transfer under the influence of excessive external pressure, the priority is to study the transfer when pressure is relieved by cutting off the root from the maternal plant.

**Figure 18:**
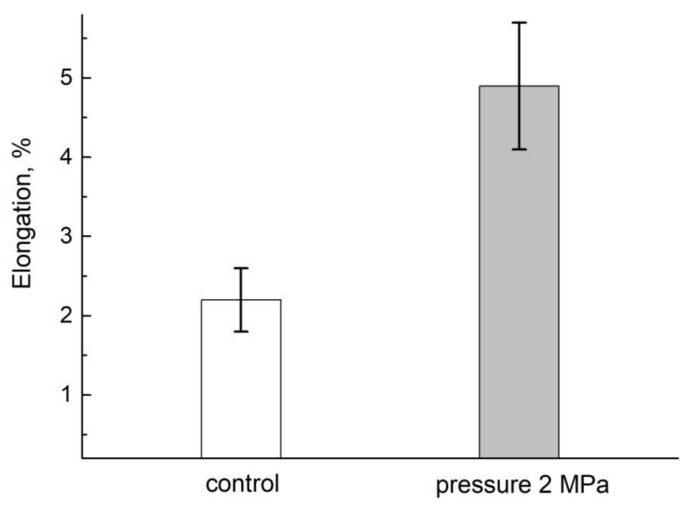
The increase (in per cent) in root segment length 4.5 h after cutting off at 23 °C and the atmosphere pressure (control) and under the treatment with the air pressure of MPa [94].

The computer dimensional analysis shows that the segment retains the growth function for 5–6 h after being cut off from the mother plant, with a decrease in the growth rate 12–20% of the norm, as shown in Figure 18 [94]:

When external air pressure is applied, the growth rate of the segments increases (up to 5% at 2 MPa). One of the reasons of the inhibition of growth when cutting off is due to a drop in pressure, which in turn causes a decrease in the concentration of air gases, dissolved in the aqueous medium of the root, and oxygen, particularly. When external air pressure is supplied to the segment, it compensates for the pressure loss and, as a result, the segment growth is accelerated.

**Figure 19:**
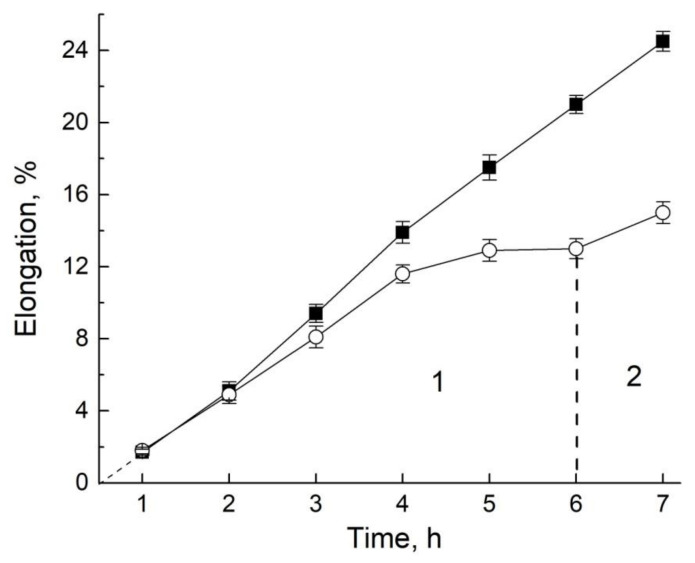
Time course of elongation (in percent) of the intact roots at 23 °C under the atmosphere pressure (solid squares) and under the impact of external air pressure of 2 MPa (open circles). After 6h of 2 MPa pressure impact (region 1) the pressure was released to the level of atmosphere (region 2) [94].

In intact roots, when pressure is applied, the growth rate, on the contrary, slows down, but tends to recover to normal after the pressure is removed, as shown in Figure 19.

It is obvious that in the hydrosystem of intact roots, normal atmospheric pressure sets the concentration of atmospheric gases dissolved in water, which provides optimal conditions for growth. When a comparatively high external pressure of up to 4 MPa is applied to the segments, 95% show a change in the transfer through the symplast of the root segment.

What happens to the water transfer when the root is cut off from the mother plant in stages?

It turned out that cutting off the leaf zone reduces the value of the permeability coefficient by an average of 10% of the control value of the root permeability of the intact sprout. The subsequent cutting off of the meristema brings the decrease to 20%. Fragmentation of the resulting segment into 3 mm sections leads to a decrease in permeability of 60%, as shown in Table 1 [94].

**Figure 20:**
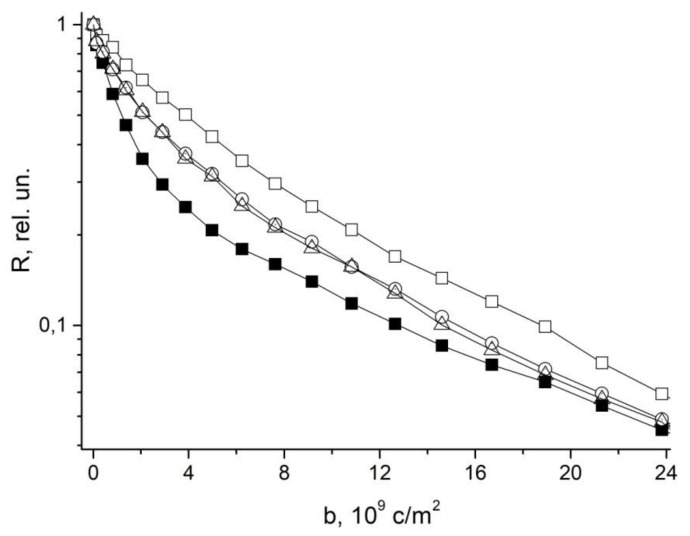
Diffusion decay for root segments: Control (solid square), 20 min treatment with sodium azide of different concentration: 0.01 M (open square), 0.001 M (open circle), 0.0005 M (open triangle) [94].

**Figure 21:**
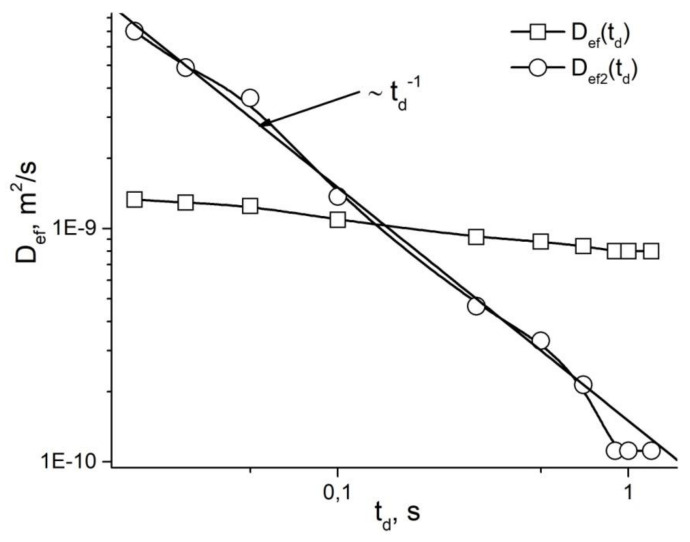
The dependence of the effective diffusion coefficient on the diffusion time for intact seedling roots (solid squares) and the result of the renormalization of *D_ef_*(*t_d_*) into the dependence *D_ef2_*(*t_d_*) as a function of *t_d_*^−1^ [94].

At the same time, the segment in the sodium azide blocker demonstrates the preservation of the ability to regulate the transfer, and in the direction of reducing the transfer, as shown in Figure 20:

The values of the effective permeability coefficients given in Table 1 for the root samples as individual parts of the plant cut off were determined from the dependence of the average effective diffusion coefficient on the diffusion time after renormalization of the *D_s_*(*t_d_*) dependence into the *D_ef_*_2_(*t_d_*) dependence as a function of $t_{d}^{-1}$. An example of renormation is given in Figure 21.

In general, the absence of dramatic changes in the intensity of intercellular water transfer in the root suction zone after it is cut off from the intact mother plant and, consequently, the exclusion of the transpiration motor of the water flow, together with the fact of preserving the ability to grow by stretching and the ability to regulate the transfer, return to the idea of the possibility of a motor for the flow of water in plants, distributed over the cells. The results of computer modeling presented in the literature indicate the possibility of aquaporins functioning as water pumps [95]. A significant decrease in permeability during fragmentation of the segment speaks in favor of blocking the transfer at the cut boundaries, which may be a protective response to prevent water loss through the cut.

### 9.2. The Influence of Changes in the Concentration of Atmospheric Gases, Namely, Carbon Dioxide, Oxygen, and Nitrogen, in the Leaf Zone of a Plant on the Transfer of Water in the Root

The fact that plants actively use atmospheric air gases in the process of metabolism suggests their influence on the functioning of the root system and, in general, on the water status of the plant. The influence of changes in the concentration of the main gases of atmospheric air, namely nitrogen, oxygen, and carbon dioxide, on water transport in the roots of intact maize plants was studied by gradient NMR [96]. Continuous monitoring of water transfer by gradient NMR was carried out with a controlled change in the concentration of gases in the leaf zone of plants placed in a chamber combined with a gradient NMR sensor [96].

**Figure 22:**
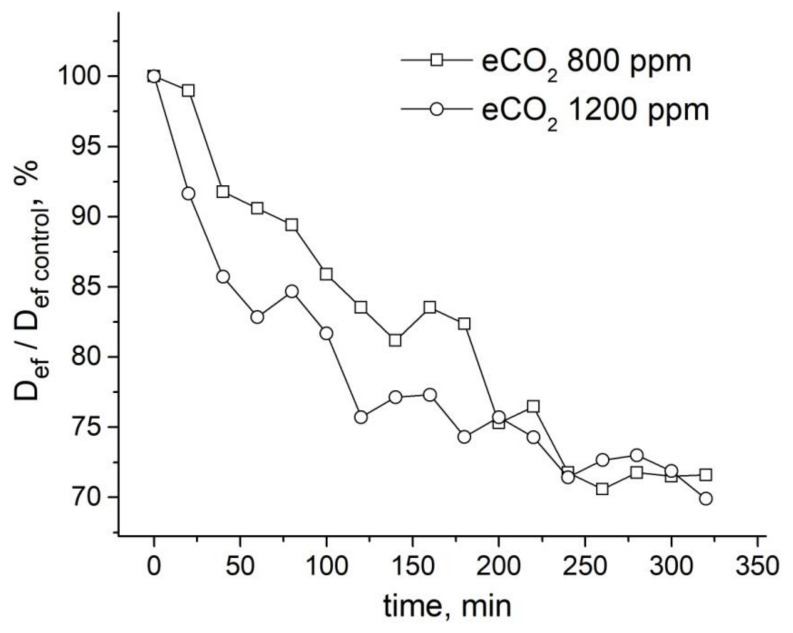
Time dependence of the ratio of effective self-diffusion coefficient (*D_ef_*) in maize roots under impact of different concentrations of CO_2_ to the control value of *D_ef_* (*D_ef control_* before CO_2_ enrichment): Open squares—dynamics of *D_ef_*/*D_ef control_* under impact of CO_2_ 800 ppm; open circles—dynamics of *D_ef_*/*D_ef control_* under impact of CO_2_ 1200 ppm [96].

An increase in CO_2_ concentration in the leaf zone of plants to 800 ppm (while the norm is 400 ppm) results in a decrease in the average effective water diffusion coefficient (D_eff_) in the roots by about 30%. A higher concentration of CO_2_ (up to 1200 ppm) results in a higher rate of Deff decrease, but the overall decrease amplitude is the same, as can be seen in Figure 22.

**Figure 23:**
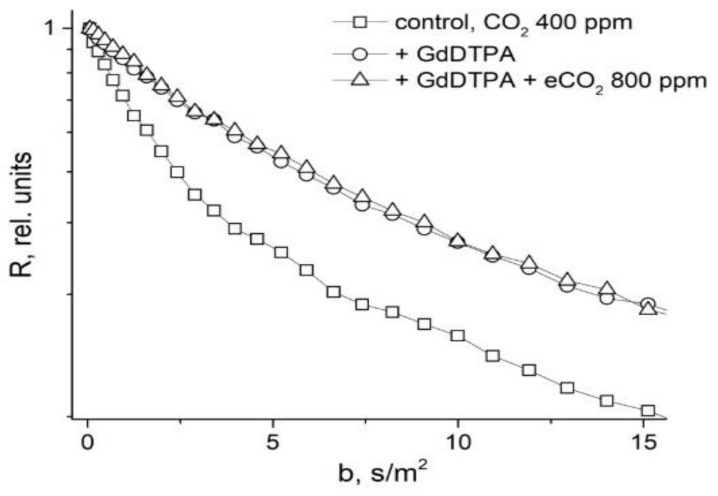
Diffusional decays for roots of intact maize plants in control (ambient CO_2_ 400 ppm) (open squares), after 2 h of roots incubation in 25 mM solute of GdDTPA (open circles) and following an increasing CO_2_ concentration to 800 ppm (open triangles), $(b={\;\gamma }^{2}\delta^{2}g^{2}t_{d}$) [96].

Surprisingly, in roots under paramagnetic doping GdDTPA, there is no reaction to an increase in CO_2_ concentration, as shown in Figure 23. Apparently, the increase in CO_2_ concentration in the leaf zone of plants does not change the transfer of water through the root symplast.

The record of DD in the roots under the blocker of aquaporins (Hg Cl_2_) and under the influence of CO_2_ showed that, along with the known fact of stomatal regulation, the aquaporins of root cells contribute to the decrease in the intensity of water transport in the roots, which is interpreted within the framework of the regulatory effect on the water permeability of aquaporins through the system of distant signaling from leaves to roots [96].

**Figure 24:**
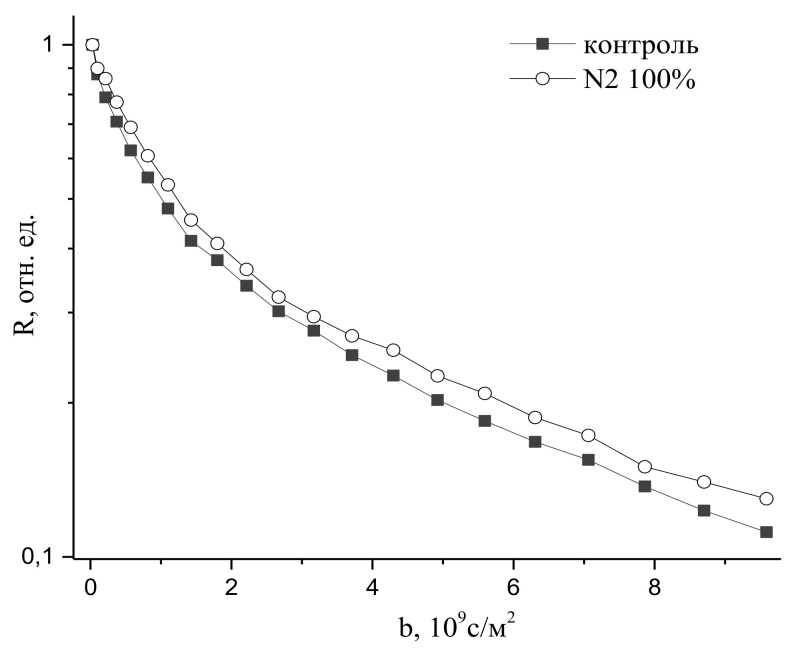
Diffusion decays of water magnetization in cells of intact corn roots in control and 2 h after increasing the nitrogen concentration N_2_ in the leaf zone up to 100% under normobaric conditions in the atmosphere ($b={\;\gamma }^{2}\delta^{2}g^{2}t_{d}$) [96].

An increase in nitrogen concentration up to 100% has only led to an insignificant decrease in water transfer, as might be expected, given the inert role of nitrogen in water metabolism, as can be seen in Figure 24.

**Figure 25:**
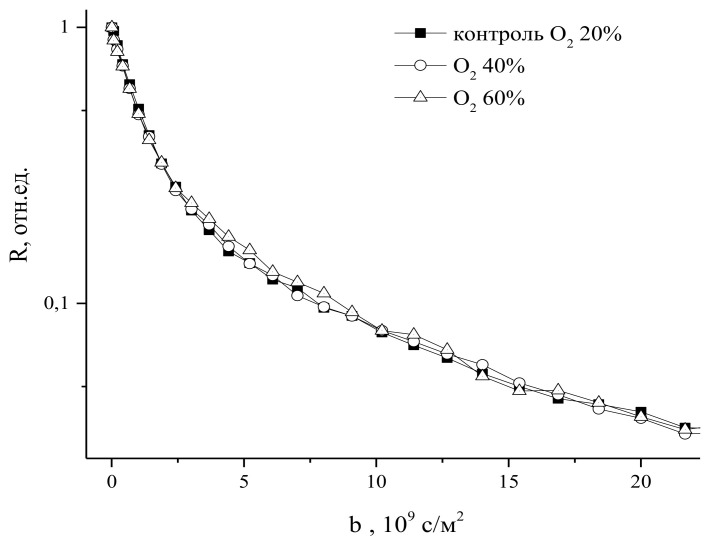
Diffusion decays of water magnetization in cells of intact corn roots in control and 30 min after an increase in the O_2_ concentration in the leaf zone up to 40% and 60% under normobaric conditions in the atmosphere ($b={\;\gamma }^{2}\delta^{2}g^{2}t_{d}$) [96].

With a decrease in oxygen concentration, a decrease in the intensity of intercellular water transport and growth of corn roots is observed, while an increase in oxygen concentration above 20% of the atmospheric norm—up to 60%—did not lead to noticeable changes in the rate of growth and intercellular water transport in the roots, as shown in Figure 25:

It is obvious that an atmospheric 20% oxygen concentration is not a limiting metabolic parameter. Moreover, an increase in oxygen concentration can lead to oxygen intoxication.

In total, the obtained results support the idea that water transfer in plants in the conditions of increased partial pressure of carbon dioxide and hypoxia (lack of oxygen) reacts by changing the functional activity and/or the number of aquaporins in cells, which begin to work to stabilize the water status under new conditions [97,98].

## 10. Conclusions

The development and application of the gradient NMR technique in studies of water transport in plants gave new information, which is difficult, and often impossible, to obtain with other methods.

The non-invasiveness of the method, sensitivity to fine movements at the atomic-molecular level, advanced methods for selective control of water flows through different transport paths, the ability to work with samples in a wide range of their structural organization from the submolecular level to intact plants, the ability to carry out measurements

Simultaneously with external influences (positive and negative pressure with a change in the composition of gases, temperature, physiologically active and membrane-active compounds, electromagnetic radiation, etc.) put this method in a special place among other methods used in biomedical studies.

As applied to the study of plants, the unique opportunities of gradient NMR were manifested in the study of water transport through the symplastic system of plants. Indeed, prior to the application of the gradient NMR technique with paramagnetic doping into the extracellular space of the tissue, there was no possibility to selectively study the diffusion transfer of water from cell to cell through the symplast or through the plasmodesmata. At one time, the lack of the possibility of experimental selection of symplast transfer forced, in the known model of composite water transport, the combination of the transmembrane and symplastic pathways into one transcellular pathway, and the authors of the MESNA model for quantitative assessment of the water flow in the radial direction of the root emphasize the urgent need for the development of methods for selective control of the transfer through the symplast. The stages of development and application of gradient NMR in studies of water transport in plants presented in this review demonstrate the nontrivial capabilities of the method and its potential for further development.

## Figures and Tables

**Table 1:** Dynamics of a decrease in root values of effective permeability coefficients during sequential cutting off of the leaf zone, meristem, and fragmentation of the root suction zone [94].

Sample Treatment	Water Permeability, m/s
Control	(6.3 ± 0.3) × 10^−5^
Excision of leaf zone	(5.7 ± 0,2) × 10^−5^
Excision of meristem zone	(4.9 ± 0.25) × 10^−5^
Fragmentation of segments into 3 mm long sections	(2.7 ± 0.3) × 10^−5^
